# CD8^+^ T cells specific for cryptic apoptosis-associated epitopes exacerbate experimental autoimmune encephalomyelitis

**DOI:** 10.1038/s41419-021-04310-6

**Published:** 2021-10-29

**Authors:** Neda Feizi, Chiara Focaccetti, Ilenia Pacella, Gloria Tucci, Alessandra Rossi, Massimo Costanza, Rosetta Pedotti, John Sidney, Alessandro Sette, Claudia La Rocca, Claudio Procaccini, Giuseppe Matarese, Vincenzo Barnaba, Silvia Piconese

**Affiliations:** 1 Department of Internal Clinical Sciences, Anesthesiology and Cardiovascular Sciences, Sapienza University of Rome, 00161 Rome, Italy; 2 Molecular Neuro-Oncology Unit, Department of Clinical Neuroscience, Fondazione IRCCS Istituto Neurologico Carlo Besta, 20133 Milan, Italy; 3 Center for Infectious Disease and Vaccine Research, La Jolla Institute for Immunology, La Jolla, CA USA; 4 Laboratorio di Immunologia, Istituto per l’Endocrinologia e l’Oncologia Sperimentale, Consiglio Nazionale delle Ricerche (IEOS-CNR), 80131 Naples, Italy; 5 Unità di Neuroimmunologia, IRCCS Fondazione Santa Lucia, 00143 Rome, Italy; 6 Dipartimento di Medicina Molecolare e Biotecnologie Mediche, Università di Napoli “Federico II”, 80131 Naples, Italy; 7 Laboratory affiliated to Istituto Pasteur Italia – Fondazione Cenci Bolognetti, 00161 Rome, Italy; 8 Present Address: Department of Human Science and Promotion of the Quality of Life, San Raffaele Roma Open University, Via di Val Cannuta 247, 00166 Rome, Italy

**Keywords:** Cell death and immune response, Immunological disorders

## Abstract

The autoimmune immunopathology occurring in multiple sclerosis (MS) is sustained by myelin-specific and -nonspecific CD8^+^ T cells. We have previously shown that, in MS, activated T cells undergoing apoptosis induce a CD8^+^ T cell response directed against antigens that are unveiled during the apoptotic process, namely caspase-cleaved structural proteins such as non-muscle myosin and vimentin. Here, we have explored in vivo the development and the function of the immune responses to cryptic apoptosis-associated epitopes (AEs) in a well-established mouse model of MS, experimental autoimmune encephalomyelitis (EAE), through a combination of immunization approaches, multiparametric flow cytometry, and functional assays. First, we confirmed that this model recapitulated the main findings observed in MS patients, namely that apoptotic T cells and effector/memory AE-specific CD8^+^ T cells accumulate in the central nervous system of mice with EAE, positively correlating with disease severity. Interestingly, we found that AE-specific CD8^+^ T cells were present also in the lymphoid organs of unprimed mice, proliferated under peptide stimulation in vitro, but failed to respond to peptide immunization in vivo, suggesting a physiological control of this response. However, when mice were immunized with AEs along with EAE induction, AE-specific CD8^+^ T cells with an effector/memory phenotype accumulated in the central nervous system, and the disease severity was exacerbated. In conclusion, we demonstrate that AE-specific autoimmunity may contribute to immunopathology in neuroinflammation.

## Introduction

Multiple Sclerosis (MS) is an autoimmune demyelinating and neurodegenerative disease of the central nervous system (CNS) [1, 2]. Experimental autoimmune encephalomyelitis (EAE) is a well-established animal model of MS [3, 4]. Dendritic cells (DCs) in brain lesions and in the cerebrospinal fluid take up antigens and migrate to the deep cervical lymph nodes (LN) for the priming or cross-priming of naïve CD4^+^ or CD8^+^ T cells, respectively [5]. CD4^+^ T lymphocytes (Th1 and Th17) are known to play dominant roles in the initiation and amplification of this disease in humans and mice. However, substantial evidence indicates that CD8^+^ T cells represent the predominant T cell population infiltrating CNS lesions of MS patients [6–8]. In addition, it has been proposed that CD8^+^ T cells can provide pathogenesis directly, by exacerbating the CD4^+^ T cell-mediated disease, or by regulating the activity of myelin-specific CD4^+^ T cells in MS [9–12], even though all the relevant (self or non-self) antigen specificities have not been yet identified. Because diverse CD8^+^ T cell activities have been similarly observed in the EAE model [13–15], it is reasonable to hypothesize that different types of CD8^+^ T cells (myelin-specific, bystander, or regulatory) can be involved in MS pathogenesis.

Our previous work has demonstrated that cryptic self-antigens, otherwise known as neo-antigens, may be generated in apoptotic activated T cells. A large number of T lymphocytes undergo apoptosis after exerting their effector function [16–18]. The proteome of apoptotic T lymphocytes comprises caspase-cleaved proteins, mostly derived from cytoskeleton, cytoplasmic, or nuclear structures (e.g., vimentin and non-muscle myosin) resulting immunogenic in conditions of chronic inflammation. Indeed, apoptotic activated T cells retain the expression of CD40L and can, thus, provide both the antigenic substrate and the danger signal for DC activation. Under these conditions, DCs phagocytose immunogenic apoptotic cells, efficiently process caspase-cleaved cellular fragmented proteins in proteasomes, and cross-present the resulting peptides on major histocompatibility complex (MHC) molecules, thus priming T cells [16–19]. These autoreactive CD8^+^ T cells can reach the main site of inflammation and perform effector functions by releasing proinflammatory cytokines and degranulating [16]. Thereafter, they undergo apoptosis as well, regenerating a vicious cycle that participates in the maintenance of a low-level inflammation and possibly perpetuating immunopathology, as demonstrated in various forms of chronic inflammatory diseases, including MS [16–18, 20–22]. The evidence that these autoreactive responses are virtually absent or functionally inefficient in normal individuals suggests that the related antigens are tolerated or ignored when expressed by live cells in their complete form, but they become immunogenic upon apoptosis, unveiling caspase-cleaved fragmented proteins potentially more susceptible to processing and cross-priming by mature DCs. The original definition of cryptic epitopes derives from data showing that only a minority of determinants on a given antigen is presented in an immunodominant manner, while the remaining peptides are silent (cryptic) [23]. We extended this concept also to those antigens that are normally hidden and are unveiled in conditions such as inflammation and tumors [24–28]. Accordingly, we found that cross-presentation of apoptotic cells (expressing the appropriate fragmented antigens), rather than cross-presentation of lysed cells (representing the proteome of live cells), induced CD8^+^ T cells specific to cryptic Apoptosis-associated Epitopes (hereinafter defined AE-specific CD8^+^ T cells) and was reduced by treating antigen-presenting cells with caspase inhibitors [16–18].

We have previously demonstrated that the AE-specific CD8^+^ responses were significantly higher and wider in MS patients than in healthy donors and displayed a phenotype of antigen-experienced T cells. Notably, the frequency of CD8^+^ T cells specific for a certain AE (e.g., non-muscle myosin, myosin heavy chain 9 [MYH9_741-749_]) was significantly higher in the cerebrospinal fluid than in the peripheral blood, and directly correlated with the disease disability score and CNS immunopathology [21].

Here, we have explored the role that the AE-specific CD8^+^ T cell response plays in vivo, taking advantage of the well-established mouse model for MS, the EAE. Our data indicate that AE-specific CD8^+^ T cells accumulate in the CNS of mice with EAE where they actively contribute to disease exacerbation.

## Results

### AE-specific CD8^+^ T cell response can be detected in the CNS of mice with EAE

**Fig. 1:**
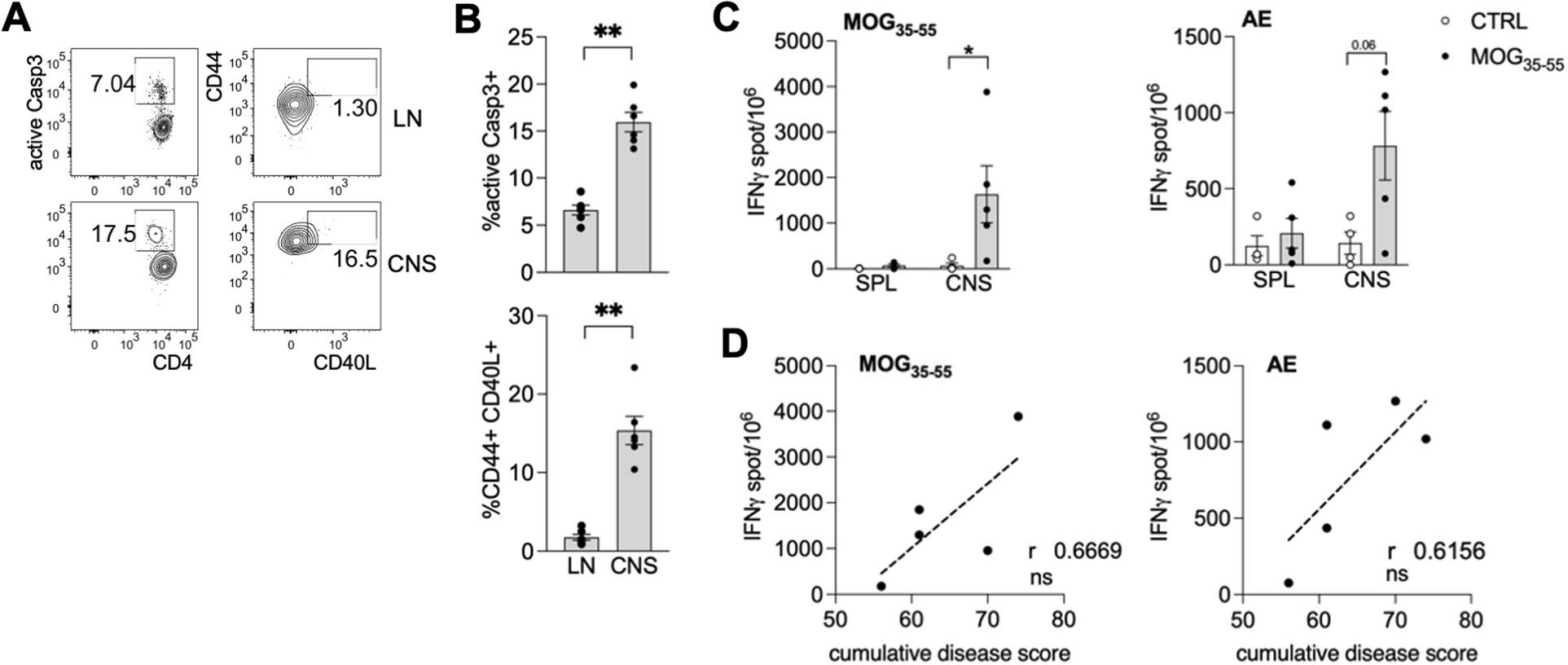
Apoptotic T cells and AE-specific CD8^+^ T cells accumulate in the CNS of mice with EAE. **A**, **B** C57BL/6 mice (*n* = 6) were immunized with 100 µg MOG_35-55_ and 400 µg heat-killed *M. tuberculosis* in IFA at day 0, and received 200 ng PTX i.v. at days 0 and 2. After 14 days, when all the mice had developed EAE, inguinal lymph nodes (LN) and brains and spinal cords (CNS) were collected, and flow cytometry analysis was performed. **A** Representative staining of active Caspase 3 in gated live single CD3^+^ CD4^+^ T cells (left) and of CD44 versus CD40L in gated apoptotic cells (right). **B** Cumulative analysis in 6 mice. Bars represent means ± SEM. **P < 0.01, by Mann–Whitney test. **C**, **D** Mice were immunized as above (*n* = 5) and sacrificed between days 28 and 48 after EAE induction. As control, not immunized (CTRL) mice were used. Lymphocytes from spleen (SPL) and central nervous system (CNS) were challenged in vitro with MOG_35-55_ or with AE-peptide pools and IFN-γ ELISpot was performed. The sum of IFN-γ spots, per 10^6^ cells, against all AE-peptide pools was calculated. **C** Cumulative analysis of IFN-γ production against MOG_35-55_ (left) or AE-peptides (right) in MOG_35-55_-immunized (*n* = 5) or control (*n* = 4) mice. Bars represent means ± SEM. *P < 0.05, by Mann–Whitney test. **D** Spearman correlation between IFN-γ response to the indicated peptides and the cumulative disease score. ns, not significant. The data are from a single experiment representative of three independent experiments.

We have previously shown a higher frequency of apoptotic T cells and AE-specific CD8^+^ T cells in the peripheral blood of MS patients compared to healthy controls [21]. Thus, we decided first to verify whether the same phenomena occurred in EAE, which is a well-established mouse model of MS that recapitulates all the major autoimmune events occurring in this disease [29]. To this aim, active EAE was induced by immunizing C57BL/6 mice with MOG_35-55_ using a conventional protocol [30]: at the acute phase of the disease, we observed that the frequency of apoptotic (active Caspase 3^+^) and activated (CD40L^+^) effector/memory CD4^+^ T cells was significantly higher in the CNS compared to inguinal lymph node as control (Fig. 1A, B).

Since the CD8^+^ T cell response was directed to HLA-A*0201-binding peptides derived from the human proteins MYH9 and VIM in MS patients [21], we focused on their murine orthologues and synthetized 70 peptides from their sequences, predicted to bind the C57BL/6 MHC-I alleles H2-Kb or H2-Db. Next, we challenged with these peptides the lymphocytes extracted from CNS (or spleen as control) of mice with post-acute EAE (from days 28 onwards), and measured IFN-γ release by ELISpot: we could detect not only MOG_35-55_-specific response, as expected, but also AE-specific response, slightly increased in the spleen, and much more in the CNS, of mice with EAE compared to controls (Fig. 1C). Interestingly, both MOG_35-55_-specific and AE-specific IFN-γ responses tended to positively correlate with the cumulative disease score in mice with EAE (Fig. 1D). Such AE-specific response was not apparent at early phases of the disease, such as at the onset of symptoms (data not shown), suggesting that it might arise following a prolonged neuroinflammation.

In order to identify the immunodominant AEs among the peptides above, we screened splenocytes from not-immunized or MOG_35-55_-immunized mice against 17 peptide pools according to a peptide matrix scheme, and selected the 5 pools that gave a higher response in MOG_35-55_-immunized compared to control mice (Figure S1A). Following matrix deconvolution, we identified 5 peptides, among which 3 were selected that ranked <1% percentile of the predicted binding for the respective MHC-I (Figure S1B-C). For these 3 peptides, namely VIM_66-74_ (SAVRLRSSV, H2-Db-restricted), MYH9_120-127_ (VINPYKNL, H2-Kb-restricted) and MYH9_761-768_ (VFFRAGVL, H2-Kb-restricted), we obtained dextramers to perform multiparametric flow cytometry-based analysis of AE-specific CD8^+^ T cells, for which a representative gating strategy is shown in Figure S2A.

**Fig. 2:**
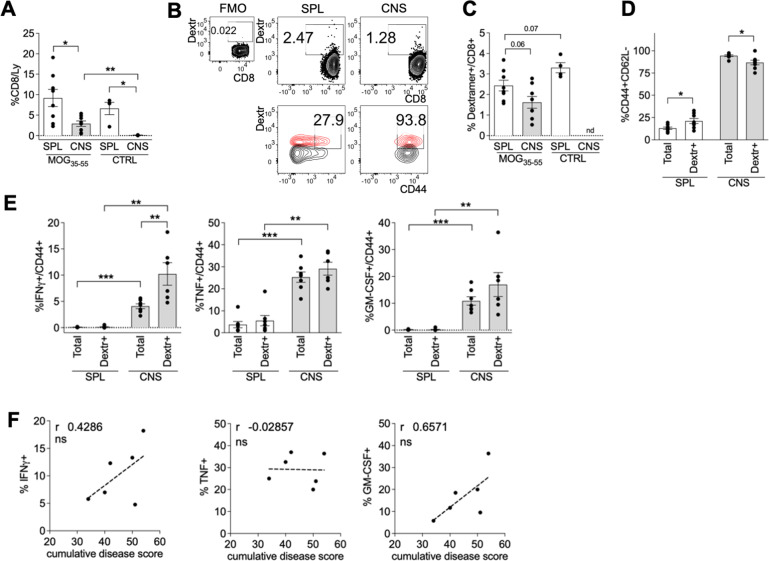
AE-specific CD8^+^ T cells display an effector/memory phenotype in the CNS of mice with EAE. C57BL/6 mice (*n* = 8) were immunized with 100 µg MOG_35-55_ and 400 µg heat-killed *M. tuberculosis* in IFA at day 0, and received 200 ng PTX i.v. at days 0 and 2. After 28 days, spleens (SPL) and brains and spinal cords (CNS) were collected, and flow cytometry analysis was performed. **A** Cumulative analysis of CD8^+^ T cell frequency in SPL and CNS of MOG_35-55_-immunized or not immunized (CTRL, *n* = 4) mice. **B** Representative stainings with pooled dextramers (Dextr) to the 3 selected peptides, VIM_66-74_ (SAVRLRSSV), MYH9_120-127_ (VINPYKNL), MYH9_761-768_ (VFFRAGVL), in gated live single CD8^+^ T cells, from SPL and CNS of mice with EAE (upper panels). CD44 expression in gated dextramer^+^ (red) or dextramer^–^ (black) CD8^+^ T cells (lower panels). **C** Cumulative analysis of dextramer^+^ cell percentage in SPL and CNS of MOG_35-55_-immunized or not immunized (CTRL, *n* = 4) mice. **D** Frequency of CD44^+^ CD62L^–^ effector/memory cells in total or dextramer^+^ CD8^+^ T cells from SPL and CNS of MOG_35-55_-immunized mice. **E** Frequency of cells producing IFN-γ, TNF or GM-CSF in gated CD44^+^ among total or dextramer^+^ CD8^+^ T cells from SPL and CNS of MOG_35-55_-immunized mice. Samples with insufficient numbers of dextramer^+^ events were not included in the analysis. **F** Spearman correlation between cytokine production in CD44^+^ dextramer^+^ cells and cumulative disease score. The data are from a single experiment representative of two independent experiments. Bars represent means ± SEM. *P < 0.05, **P < 0.01, ***P < 0.005, by Mann-Whitney test. nd, not determined. ns, not significant.

CD8^+^ T cells infiltrated the CNS of mice with EAE, being virtually absent in the CNS of control mice, as expected (Fig. 2A). Notably, both the spleen and the CNS of mice with EAE contained AE-specific CD8^+^ T cells, as detected by pooled dextramer staining against the three specificities (Fig. 2B, C). Dextramer^+^ CD8^+^ T cells contained more effector/memory cells than total CD8^+^ T cells in the spleen, while both dextramer^+^ and total CD8^+^ T cells were mostly effector/memory in the CNS (Fig. 2B, D). An extensive phenotypic analysis revealed that AE-specific CD8^+^ T cells resembled total CD8^+^ T cells and presented a CD44^+^ CD62L^-^ CD127^int^ PD1^+^ Tbet^+^ Eomes^+^ phenotype, typical of terminally differentiated effector/memory CD8^+^ T cells, in the CNS of mice with EAE (Figure S2B). When cells were shortly restimulated ex vivo, both total and AE-specific effector/memory CD8^+^ T cells produced cytokines such as IFN-γ, TNF and GM-CSF at higher levels in the CNS than in the spleen, being IFN-γ also more strongly produced by dextramer^+^ compared to total CD8^+^ T cells (Fig. 2E). Interestingly, the percentage of dextramer^+^ effector/memory CD8^+^ T cells producing IFN-γ or GM-CSF (but not TNF) showed a trend for a positive correlation with the cumulative disease score (Fig. 2F). Overall, these data indicate that the apoptosis of activated T cells and AE-specific CD8^+^ response can be observed in the affected tissue in the EAE model.

### AE-specific CD8^+^ T cells are present in not-immunized mice

**Fig. 3:**
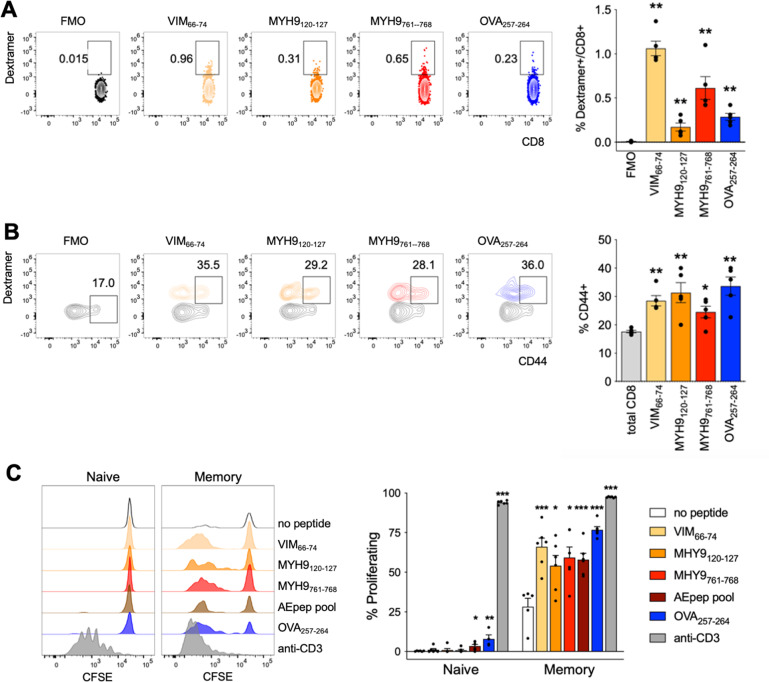
AE-specific CD8^+^ T cells can be found in naïve mice. **A**, **B** Splenocytes from C57BL/6 naïve mice (*n* = 5) were analyzed by flow cytometry. **A** Representative stainings (left) and cumulative analysis (right) of CD8^+^ T cells specific for the indicated single peptides. FMO, fluorescence-minus-one control. *P < 0.05, **P < 0.01, by Mann–Whitney test, compared to the FMO. **B** Representative stainings (left) and cumulative analysis (right) of CD44^+^ cells in the shown subsets. *P < 0.05, **P < 0.01, by Mann–Whitney test, compared to the total CD8^+^ T cells. Overall this analysis was repeated thrice. **C** CFSE profiles (left) and percentages of proliferating cells (right) in gated naïve or memory CD8^+^ T cells that have been stimulated for 4 days with irradiated splenocytes, anti-CD28, plus the indicated stimuli. The data are from a single experiment representative of three independent experiments. Bars represent means ± SEM. *P < 0.05, **P < 0.01, ***P < 0.005, by Mann-Whitney test, compared to “no peptide” in each subgroup.

The data collected so far indicate that not-immunized mice may contain AE-specific CD8^+^ T cells. To investigate this aspect in more detail, we analyzed the frequency and the phenotype of AE-specific CD8^+^ T cells in the peripheral lymphoid organs of naïve C57BL/6 mice through single dextramer staining and multiparametric flow cytometry. We found that CD8^+^ T cells in the spleen contained detectable proportions of cells specific for any of the three tested AE specificities; interestingly, we could reveal also CD8^+^ T cells specific for the OVA_257-264_ control peptide, in line with previous data in the literature showing that unprimed mice contain antigen-specific CD8^+^ T cells with a memory-like phenotype [31] (Fig. 3A). According to those results, AE-specific CD8^+^ T cells were relatively enriched in CD44^+^ effector/memory T cells, compared to total CD8^+^ T cells (Fig. 3B). When we isolated CD44^–^ and CD44^+^ CD8^+^ T cells from the spleen of naïve mice, we found that both subsets readily proliferated in vitro in response to a polyclonal stimulus; however, only CD44^+^ CD8^+^ T cells responded not only to OVA peptide but also to all the tested AEs, either single or pooled (Fig. 3C), thus demonstrating that memory CD8^+^ T cells of unprimed mice contain AE-responsive cells. These cells probably develop in the thymus from positive selection to cryptic peptides locally provided by apoptotic thymocytes: indeed, we could detect AE-specific, but not OVA_257-264_-specific, cells, among both double positive and CD8^+^ single positive thymocytes of naïve mice (Fig. S3).

**Fig. 4:**
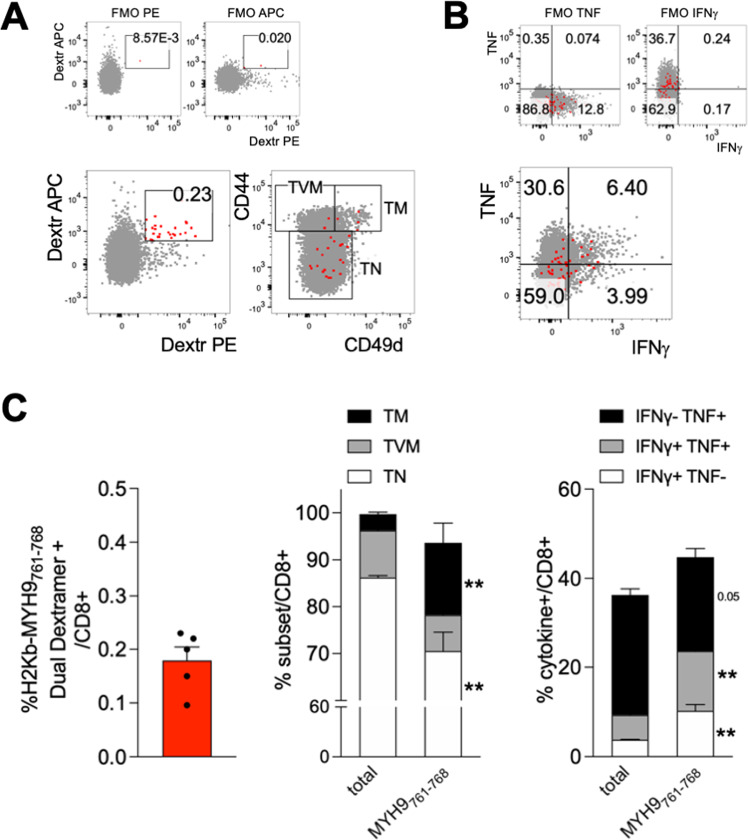
Dual-dextramer staining reveals AE-specific CD8^+^ T cells have a memory phenotype and produce cytokines in naïve mice. Splenocytes from C57BL/6 naïve mice (*n* = 5) were analyzed by flow cytometry. **A** Representative dual dextramer staining (left) and CD44/CD49d staining (right) in total (gray) or antigen-specific (red) CD8^+^ T cells. T_VM_, T virtual memory; T_M_, T memory; T_N_, T naïve. **B** Representative cytokine staining in total (gray) or antigen-specific (red) CD8^+^ T cells. **C** Cumulative analyses of dual dextramer^+^ cells, memory subsets and cytokine producing cells in total compared to antigen-specific CD8^+^ T cells. The data are from a single experiment representative of two independent experiments. Bars represent means ± SEM. **P < 0.01, by Mann–Whitney test, compared to total cells.

Next, we wondered if the effector/memory AE-specific CD8^+^ T cells from naïve mice were conventional memory cells (T_M_) or could be part of the so-called virtual memory CD8^+^ T cells (T_VM_), which are antigen-inexperienced memory-like cells developing in unprimed mice in response to cytokine stimulation and recognizing self-antigens with high affinity [32]. Therefore, we analyzed the phenotype of AE-specific CD8^+^ T cells in naïve mice, to determine whether they were enriched in T_VM_ or T_M_, based on the expression of the α4 integrin CD49d which distinguishes these two subsets [31]. In order to refine our analysis and to improve specificity, we focused on a single AE specificity (MYH9_761-768_) and performed dual dextramer staining for that peptide. Through this approach, we could determine that the frequency of MYH9_761-768_ was below 0.5% in the spleen of naïve mice, and that they were significantly enriched not in T_VM_ but in T_M_ cells, as assessed by CD49d staining (Fig. 4A–C). Notably, MYH9_761-768_-specific CD8^+^ T cells contained significantly higher frequencies of cells producing IFN-γ and/or TNF following a short non-antigen-specific restimulation ex vivo, compared to total CD8^+^ T cells (Fig. 4B, C). However, we could not detect cytokine production following cognate peptide stimulation (not shown). These results indicated that AE-specific CD8^+^ T cells were likely antigen-experienced, conventional memory cells with competence for bystander cytokine production, but unable to perform an antigen-dependent cytokine response.

**Fig. 5:**
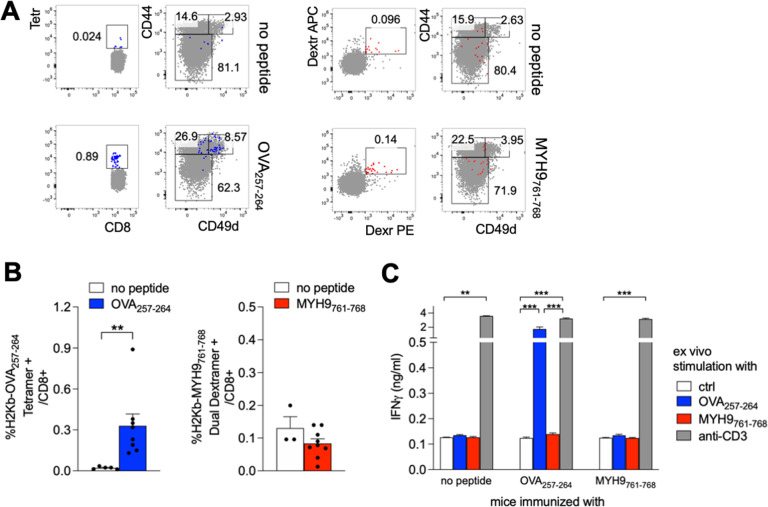
Immunization with OVA_257-264_, but not with MYH9_761-768_, induces antigen-specific CD8^+^ T cell responses. C57BL/6 mice were immunized with OVA_257-264_ (*n* = 8), with MYH9_761-768_ (*n* = 8) or with no peptide (*n* = 5), and flow cytometry analysis was performed. Representative stainings (**A**) and percentages (**B**) of OVA_257-264_-specific (blue) or MYH9_761-768_-specific (red) cells in mice immunized with the respective peptides or with saline (no peptide), in alum and MPL as adjuvants. Samples with insufficient numbers of dextramer^+^ or tetramer^+^ events were not included in the analysis. **C** Splenocytes were challenged ex vivo for 48 h with the indicated stimuli and IFN-γ was measured in the supernatants through ELISA. The data are from a single experiment representative of two independent experiments. Bars represent means ± SEM. **P < 0.01, ***P < 0.005, by Mann–Whitney test.

Since AE-specific memory CD8^+^ T cells from naïve mice readily proliferated in vitro in response to the respective peptides (Fig. 3C), we sought to verify whether they were also responsive in vivo, in a setting of peptide-based immunization. To this aim, we adopted a protocol that was previously shown to induce effective CD8^+^ T cell responses, that is the repeated subcutaneous administration of the peptide admixed with aluminum (alum) and monophosphoryl lipid A (MPL) as adjuvants [33]. When mice were immunized with the OVA_257-264_ peptide, a clear expansion of antigen-specific CD8^+^ T cells, mostly showing a CD44^+^ CD49d^+^ T_M_ phenotype, could be detected in the spleen; conversely, immunization with MYH9_761-768_ peptide did not elicit any increase in antigen-specific CD8^+^ T cells, as measured through dual dextramer-straining, or any change in T_M_ cells, compared to mice receiving adjuvants alone as control (Fig. 5A, B). In line with these findings, splenocytes from OVA_257-264_-immunized mice but not from MYH9_761-768_-immunized mice released IFN-γ when challenged ex vivo with the respective peptide, as assessed through ELISA (Fig. 5C). Altogether, these data demonstrate that AE-specific CD8^+^ T cells with a memory phenotype develop in naïve mice, proliferate in vitro when stimulated with the cognate antigen, but do not respond to peptide immunization in vivo.

### AE-specific CD8^+^ T cell response exacerbates EAE severity

**Fig. 6:**
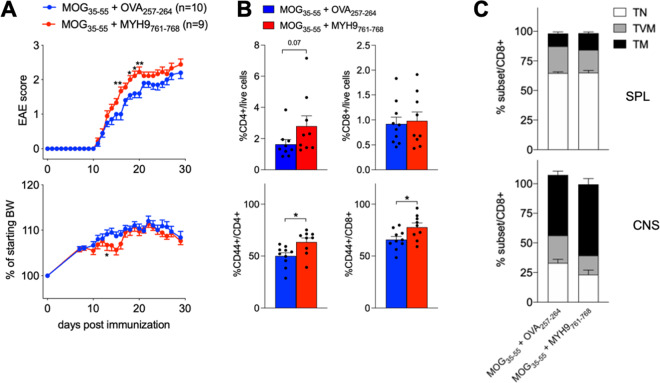
MYH9_761-768_ immunization exacerbates EAE disease course. C57BL/6 mice were immunized with 100 µg MOG_35-55,_ admixed with OVA_257-264_ (*n* = 10) or with MYH9_761-768_ (*n* = 9), and received 200 ng *B. pertussis* Toxin (PTX) i.p. at days 0 and 2. **A** EAE score and changes in body weight were monitored. *P < 0.05, **P < 0.01, by Mann–Whitney test. **B, C** Mice were sacrificed at day 29 and flow cytometry analysis was performed in spleens (SPL) and central nervous system (CNS). **B** Percentages of total CD4^+^ and CD8^+^ T cells (upper panels), and of CD44^+^ CD4^+^ or CD8^+^ T cells (lower panels), in the CNS of mice immunized with MOG_35-55_ plus OVA_257-264_ (blue) or plus MYH9_761-768_ (red). *P < 0.05, by Mann–Whitney test. **C** Percentages of T naïve (T_N_), T virtual memory (T_VM_), and T memory (T_M_) in gated CD8^+^ T cells from SPL or CNS of mice immunized with MOG_35-55_ plus OVA_257-264_ or plus MYH9_761-768_. The data are from a single experiment representative of two independent experiments. Bars represent means ± SEM.

Our data demonstrate that AE-specific CD8^+^ T cells accumulate in the CNS of mice with EAE and of MS patients, positively correlating with indexes of disease severity [21]. To finally support the conclusion that these cells contribute to immunopathology in neuroinflammation, we induced active EAE by coimmunizing mice with MOG_35-55_ plus MYH9_761-768_ peptides, or MOG_35-55_ plus OVA_257-264_ as control. MYH9_761-768_ coadministration induced a significant increase in the EAE score, which was accompanied by a weight loss around day 15 after immunization (Fig. 6A). The disease exacerbation in MYH9_761-768_-coimmunized mice was associated to a higher infiltration in the CNS not of the total CD4^+^ or CD8^+^ T cells, but of their CD44^+^ effector/memory subsets (Fig. 6B). Interestingly, CD8^+^ T cells comprised mostly T_M_, and only a minority of T_VM_, in the CNS of mice with EAE, irrespectively of the co-administered peptide (Fig. 6C).

**Fig. 7:**
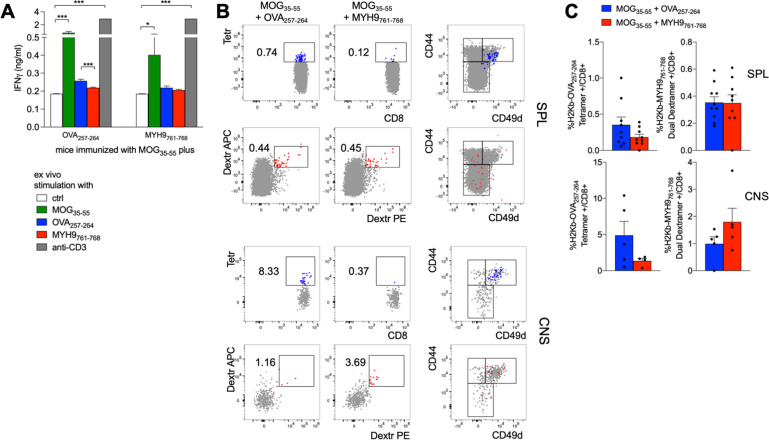
MYH9_761-768_ immunization enhances antigen-specific response in the CNS of mice with EAE. C57BL/6 mice, immunized with 100 µg MOG_35-55,_ admixed with OVA_257-264_ (*n* = 10) or with MYH9_761-768_ (*n* = 9), were sacrificed at day 29. **A** Splenocytes were challenged ex vivo for 48 hr with the indicated stimuli and IFN-γ was measured in the supernatants through ELISA. Bars represent means ± SEM. *P < 0.05, ***P < 0.005, by Mann–Whitney test. **B** Representative stainings of OVA_257-264_-specific (blue) or MYH9_761-768_-specific (red) cells in CD8^+^ T cells from SPL or CNS of mice immunized with MOG_35-55_ plus the indicated peptides. Right plots show CD44/CD49d expression in gated antigen-specific or total (gray) cells. **C** Cumulative analysis of the percentage of OVA_257-264_-specific (left plots) or MYH9_761-768_-specific (right plots) cells in CD8^+^ T cells from SPL or CNS of mice immunized with MOG_35-55_ plus OVA_257-264_ (blue) or MYH9_761-768_ (red). The data are from a single experiment representative of two independent experiments. Bars represent means ± SEM.

In mice coimmunized with MOG_35-55_ and MYH9_761-768_ peptides, we failed to reveal in the spleen an MYH9_761-768_-specific effector response, while an OVA_257-264_-specific response was detectable in control mice, as measured through IFN-γ ELISA in the supernatants of splenocytes restimulated ex vivo with cognate peptide (Fig. 7A). Accordingly, OVA_257-264_ but not MYH9_761-768_ induced the expansion of antigen-specific CD8^+^ T cells, showing a T_M_ phenotype, in the spleen of coimmunized mice (Fig. 7B, C). Notably, we could observe that MYH9_761-768_-specific CD8^+^ T cells, with a T_M_ phenotype, did in fact accumulate in the CNS of MYH9_761-768_-coimmunized mice, compared to controls (Fig. 7B, C). These data demonstrate that, in a setting of neuroinflammation, MYH9_761-768_-specific CD8^+^ T cells respond to antigen stimulation in vivo, migrate into the affected tissue and promote disease exacerbation.

## Discussion

Compared to CD4^+^ T cells, the role of myelin-specific and bystander CD8^+^ T cells in autoimmune neuroinflammation is more elusive. Here we have shown that, in EAE, cryptic antigens, possibly unveiled during the apoptotic process, elicit a spreading of CD8^+^ T cell response that exacerbates the disease course.

Previous data demonstrated that myelin-specific CD8^+^ T cells are present in naïve mice, are activated during EAE, and contribute to disease pathogenesis [13, 34]. DCs that are responsible for the cross priming of encephalitogenic CD8^+^ T cells are the so-called Tip-DC, defined by the expression of TNF and inducible nitric oxide synthase, and derived from inflammatory monocytes [5]. Interestingly, myelin-specific CD8^+^ T cells exacerbated inflammation more in the brain than in the spinal cord, promoting ROS release by inflammatory monocytes and DCs [35]. However, most of the clonally expanded CD8^+^ T cells extracted from the CNS of mice with EAE did not respond to myelin antigens [36]. Some of these antigens were responsible for the activation of regulatory CD8^+^ T cell populations, which suppressed encephalitogenic CD4^+^ T cells and thus induced a milder EAE course [36]. Using a very similar experimental approach (the co-immunization with MOG_35-55_ together with an MHC-I-restricted peptide), we found that the MYH9_761-768_ did not attenuate but rather exacerbated the disease severity, promoting a higher infiltration into the CNS of CD44^+^ CD4^+^ and CD8^+^ T cells. This finding was in line with the positive correlations observed between AE-specific CD8^+^ T cell frequency/functions in the CNS and the severity of EAE (this study) and MS [21]. In the CNS of mice with EAE, the phenotype of AE-specific CD8^+^ T cells was fully comparable to that of polyclonal CD8^+^ T cells, both resembling terminal effector cytokine-producing T cells. Therefore, we have identified a population of not myelin-specific CD8^+^ T cells that amplify immunopathology in this model.

We found AE-specific CD8^+^ T cells also in the lymphoid organs of naïve wild-type mice. Antigen-inexperienced CD8^+^ T cells with a memory phenotype have been described in the lymphoid organs of unprimed mice. These cells are characterized by the low expression of CD49d, and can be distinguished into two subsets, namely the innate and the virtual memory CD8^+^ T cells [32]. According to previous data [31], we found that the CD44^+^ compartment of CD8^+^ T cells contained cells that proliferated in response to an exogenous antigen, OVA_257-264_, to which mice had not been previously exposed. Interestingly, AEs elicited a similar outcome, prompting us to hypothesize that AE-specific CD8^+^ T cells in unprimed mice may be comprised among T_VM_ cells. However, the analysis of CD49d expression revealed that AE-specific CD8^+^ T cells included naïve as well as memory CD8^+^ T cells. This finding is in line with our recent data describing the expansion of human AE-specific CD8^+^ T cells, with a mixed phenotype, in patients with chronic HBV infection and fibrosis [22].

Notably, in naïve mice, AE-specific CD8^+^ T cells were relatively enriched in memory (CD49d^+^), rather than virtual memory (CD49d^–^), CD8^+^ T cells. This result suggests that AE-specific CD8^+^ T cells may comprise antigen-experienced cells in naïve mice that can potentially be activated in a bystander fashion by cytokines or contact signals in inflammatory conditions. AE-specific CD8^+^ T cells may derive from the homeostatic T cell proliferation, a key mechanism requiring a precise balance between proliferation and apoptosis, through which naïve T cells generate a constant number of memory phenotype cells with increasing T cell repertoire complexity, including cross- or auto-reactive T cells [37]. An alternative, but not mutually exclusive, possibility is that mice are exposed to the same antigens that are contained in homolog proteins expressed by commensal eukaryotic species. Indeed, all the three tested AEs (VIM_66-74_, MYH9_120-127_ and MYH9_761-768_) are conserved in several genera of fungi including commensals like *Aspergillus* (not shown). Therefore, it is possible that commensal exposure may prime AE-specific CD8^+^ T cells in naïve mice.

Another possible explanation is that these cells recognize in naïve mice those myosin- and vimentin-derived antigens produced during the physiological apoptosis of a variety of cells, including T cells. This event may occur in establishing both central and peripheral tolerance. In the thymus, the abundant apoptotic thymocytes may represent a source of antigen for the positive selection of AE-specific T cells. In the periphery, in naïve mice, AE-specific CD8^+^ T cells may recognize their cognate antigens derived from the physiological apoptosis of tissue and immune cells, however they would receive a suboptimal and/or abortive priming. Indeed, several data in experimental models demonstrate that apoptotic cells are generally captured by phagocytes that induce a non-inflammatory clearance and a tolerogenic response [38]. Apoptotic cells have been shown to promote tolerance by inducing “helpless” CD8^+^ T cells, which release TRAIL and eliminate activated T cells [39], and which are characterized by defective recall responses [40, 41]. Therefore, it is reasonable to hypothesize that AE-specific CD8^+^ T cells in naive mice are primed in a physiological and tolerogenic fashion by dying cells, are probably helpless memory cells, and are refractory to conventional recall response with peptide immunization in vivo unless they are exposed to severe tolerance subversion in neuroinflammation.

Apoptosis can become immunogenic in certain contexts, depending on the nature of the dying cell, the type of death stimulus and pathway, the location of death, and the responding immune cells [19, 42]. In the case of dying T cells, their activation and CD154 upregulation, prior to apoptosis induction, abolishes their tolerogenic function in both human immunopathology contexts [18] and experimental models [43]. We show here a significant accumulation of apoptotic CD154^+^ T cells in the CNS of mice with EAE. One of the mechanisms sustaining activated T cell death in that context could be activation-induced cell death (AICD), following secondary antigen encounter by encephalitogenic T cells at the site of tissue damage. Fas-mediated AICD has been shown to occur in EAE in response to high affinity peptide ligands and to play a regulatory role promoting clinical remission [44, 45]. Our results indicate that a potentially protective mechanism may turn into a detrimental event, as activated apoptotic T cells become an immunogenic source of cryptic self-antigens.

Under normal conditions, these autoreactive T cells are maintained ignorant or tolerant by a large series of regulatory mechanisms, including those provided by regulatory T cells (Tregs), which can be subverted in conditions of severe or chronic inflammation [46, 47]. In line with this hypothesis, AE-specific CD8^+^ T cells, contrary to those OVA_257-264_-specific, were not responsive to peptide immunization in vivo in the presence of MPL/alum adjuvants in naïve mice. Instead, these cells were activated and accumulated in the CNS when peptide administration was performed along with active EAE induction. In this setting of neuroinflammation, characterized by accumulation of apoptotic T cells in the CNS, AE-specific CD8^+^ T cells likely responded to antigen-stimulation in vivo, produced inflammatory cytokines, migrated into the inflamed brain, and exacerbated the neurologic disease. In this setting, we could not formally demonstrate a causal link between higher T cell apoptosis and increased AE-specific CD8 response; however, we have previously shown in a human setting that myosin- and vimentin-specific CD8^+^ T cell responses were triggered by apoptotic cells in vitro in a caspase-dependent fashion [18]. Based on those data, we can speculate that AE-specific CD8^+^ T cell activation may be induced in the CNS by increased apoptosis of activated T cells. This interpretation is compatible with the cryptic antigen model, whereby the self-antigens are processed and presented in an altered fashion in the diseased tissue [48]: indeed, structural proteins such as myosin and vimentin could be efficiently fragmented only by caspases in apoptotic cells, thus generating immunogenic epitopes [18].

Despite been partially antigen-experienced, AE-specific CD8^+^ T cells are normally ignorant (because specific to hidden antigens, such as those unveiled as caspase-cleaved antigens during apoptosis) or kept under control by cell-intrinsic or -extrinsic mechanisms of immune tolerance. By contrast, such AE-specific CD8^+^ T cell unresponsiveness could be subverted not through a simple immunization, but only in a context of autoimmune tissue inflammation, which is characterized by multiple Treg dysfunctions [49]. Accordingly, we have previously published that, in human autoimmune disease, the differentiation of AE-specific CD8^+^ T cells is bidirectionally modulated by Tregs, according to the disease severity and the degree of CD8^+^ T cell effector differentiation [20]. The spreading of these responses can amplify immunopathology, likely as a consequence of a primary myelin-specific autoimmune or pathogen-triggered (e.g., by viral infections [50] or gut microbiota [51, 52]) immune response initiating EAE or MS and providing the immunogenic apoptotic substrate [20].

Finally, our data pave the way for testing if innovative therapeutic strategies with inhibitory checkpoint agonists [53], or novel therapeutic compounds targeting molecules involved in MS immunopathology [54], may provide beneficial effects in autoimmunity, because they may dampen not only T cell activation, but also the consequent immunogenic apoptosis that may provide a huge plethora of cryptic AEs and thus perpetuates the chronic immune activation.

## Materials and methods

### Mice

C57BL/6 mice were purchased from Charles River Laboratories (Calco, Italy). Age-matched female or male mice, 7- to 12-weeks old, were used in all experiments. Mice were maintained under pathogen-free conditions at the animal facility of Dipartimento di Scienze Anatomiche, Istologiche, Medico legali e dell’Apparato locomotore (SAIMLAL), in Sapienza Università di Roma. All animal procedures were carried out according to ethical guidelines for the use of animal samples, in line with European and national laws and approved by the institutional committee and by the Italian Ministry of Health (authorization no. 481/2015-PR).

### Peptide synthesis

MOG_35–55_ peptide (MEVGWYRSPFSRVVHLYRNGK) was synthesized using standard 9-fluorenylmethoxycarbonyl chemistry on a 433 A automated peptide synthesizer (Applied Biosystems) and purified by HPLC (purity was >95%).

The H2-Kb or H2-Db candidate peptides studied were derived from the murine MYH9 (Uniprot Q8VDD5) and VIM (Uniprot P20152) proteins and were selected from the top 1% scoring peptides of each protein using a consensus of the SMM and ANN algorithms [55, 56]. Predictions were performed considering two different peptide lengths (8- and 9-mers for Kb, and 9- and 10-mers for Db), reflecting canonical ligand lengths for the respective MHC alleles. The list of the obtained 70 peptides synthesized is contained in Table S1. The 3 selected peptides VIM_66-74_ (SAVRLRSSV), MYH9_120-127_ (VINPYKNL), MYH9_761-768_ (VFFRAGVL) and control peptide OVA_275-282_ (SIINFEKL) were synthesized by Primm Biotech (Italy) and provided at > 95% purity as verified by HPLC and mass spectrometry analysis. Peptides were dissolved in sterile water, diluted to 20 mg/ml and frozen at −20 °C until required.

### EAE experiments

For the induction of conventional EAE, naïve female C57BL/6 mice, 8-12 weeks old, were injected subcutaneously (s.c.) in their flanks with 0.1 ml of an emulsion containing 100 μg of MOG_35–55_ and 400 μg heat-killed *M. tuberculosis* (H37Ra, Difco) in Incomplete Freund’s adjuvant (IFA, Difco) and, the same day and 2 days later, received intravenously (i.v.) 200 ng of *B. pertussis* Toxin (PTX) (List Laboratories).

In co-immunization experiments, naïve female C57BL/6 mice, 8-12 weeks old, were injected s.c. in each flank with 0.1 ml of an emulsion containing 100 μg of MOG_35-55_ and MYH9_761-768_ peptide (100 μg), or OVA_275-282_ peptide (100 μg), plus 400 μg heat-killed *M. tuberculosis* (H37Ra, Difco) in IFA and, the same day and 2 days later, received i.p. 200 ng of *B. pertussis* Toxin (Sigma Aldrich). 28 days later, mice were sacrificed and mononuclear cells from spleen and CNS (brain and spinal cord) were harvested for further analyses.

Mice were weighed daily and assessed daily for neurological signs of EAE according to the following 5-point scale: 0, healthy; 1, tail weakness or paralysis; 2, paraparesis (incomplete paralysis of one or two hind limbs/plegia of one hind limb); 3, paraplegia extending to the thoracic level; 4, forelimb weakness or paralysis with hind limbs paraparesis or paraplegia; and 5, moribund or dead animal.

Mice were maintained under specific pathogen-free conditions at the animal facility of Fondazione IRCCS Istituto Neurologico “Carlo Besta” in Milan, or at the animal facility of Dipartimento di Medicina Molecolare e Biotecnologie Mediche, Università di Napoli “Federico II” in Naples. All procedures involving animals were approved by the institutional ethical committees and by the Italian General Direction for Animal Health at the Ministry of Health. Animal studies were performed in accordance with the institutional guidelines and national law (DL116/92) and carried out according to the Principles of Laboratory Animal Care (European Communities Council Directive 2010/63/EU), in order to minimize discomfort for animals.

### AE immunization in MPL/alum

Naïve female C57BL/6 mice, 7 weeks old, were injected s.c. in their flanks with 0.1 ml of an emulsion with MYH9_761-768_ peptide (100 μg), or OVA_275-282_ peptide (100 μg), mixed with 20 μg MPL (Monophosphoryl Lipid A) Adjuvant (InvivoGen) plus Alum (Aluminum hydroxide 2%), (Alum to peptide solution, 1:1 volume ratio, InvivoGen). Immunization was performed on days 0, 14, and 28. Mice were sacrificed two weeks after the last boost, and mononuclear cells were extracted from spleens for further analyses.

### Tissue processing and T cell extraction

Lymph nodes and spleens were passed through a 70-μm cell strainer to obtain single-cell suspension. Erythrocytes were lysed with 4 min incubation with ACK lysis solution (Gibco) at 4 °C. To extract mononuclear cells from CNS, brain and spinal cord were passed through a 70-μm cell strainer in ice-cold PBS 2% FBS; next, cells were centrifuged, and cell pellets were resuspended in 4 ml of 37% Percoll (GE Healthcare) at room temperature, overlaid onto 70% Percoll, and centrifuged at 1600 rpm for 20 min without brake; finally, infiltrating lymphocytes were collected from the interface between 37% and 70% Percoll solutions.

### Flow cytometry

In all experiments, dead cells were excluded after labeling with eFluor780 Fixable viability dye (Thermofisher Scientific). Surface staining was performed incubating the cells in PBS 2% FBS for 20 min at 4 °C, with combinations of the following antibodies: CD44 PE-Cy7 (Biolegend, cat. 103030), CD44 BV510 (BD Biosciences, cat. 563114), CD40L APC (Biolegend, cat. 106509), active Caspase 3 V450 (BD Biosciences, cat. 560627), CD3 BV510 (Biolegend, cat. 100233) or PE-Cy7 (Biolegend, cat. 100220) or BB700 (BD Biosciences, cat. 566494), CD4 BV605 (Biolegend, cat. 100548), CD8 BV785 (Biolegend, cat. 100750) or FITC (Clone KT15, MBL, cat. D271-4), CD49d PE (BD Biosciences, cat. 553157) or BV605 (BD Biosciences, cat. 740341), CD62L PE-CF594 (Biolegend, cat. 104448) or AF700 (Biolegend, cat. 104426), B220 APC-H7 (BD Biosciences, cat. 565371), PD1 PE (Biolegend, cat. 135205), CD127 PerCP-Cy5.5 (Biolegend, cat. 135022), Tbet BV711 (Biolegend, cat. 644819), Eomes PE-Cy7 (Thermofisher Scientific, cat. 25-4875-80).

For antigen-specific T cell detection, after viability dye staining, cells were incubated with APC- or PE-conjugated dextramers (Immudex, cat. JD4156) or tetramer (iTagTM MHC Tetramer, MBL, cat. TB-5001-2), at room temperature for 20 min, then stained for surface markers (without previously washing the multimers).

To analyze cytokine production by intracellular staining, cells were first incubated with Protein Transport Inhibitor cocktail (500x) (Thermofisher Scientific), or with Cell stimulation plus Protein Transport Inhibitor Cocktail (500x) as a positive control (Thermofisher Scientific), for 4 hr at 37 °C. Cells were washed, stained for surface markers, fixed and permeabilized using Cytofix/Cytoperm solution (BD Biosciences) at 4 °C for 20 min, washed with Perm/wash Buffer (BD Biosciences) and stained with TNF PE-Cy7 (Biolegend, cat. 506324) and IFN-γ BV711 (Biolegend, cat. 505835) or BV421 (Biolegend, cat. 505830) for 20 min at 4 °C. For intranuclear staining, cells were stained with Foxp3/Transcription Factor Staining Buffer Set according to manufacturer’s instruction (Thermofisher Scientific).

In all experiments, cells were acquired with LSR Fortessa cytometer (Becton Dickinson) and analyzed with FlowJo software version 10 (TreeStar).

### In vitro T cell proliferation

Splenocytes were collected from naïve C57BL/6 female mice. CD8^+^ naïve and memory T cells were isolated with Naïve CD8a^+^ T cell isolation kit (Miltenyi Biotec), following the manufacturer’s instructions. CD8^–^ cells from splenocytes were used after γ-irradiation (3000 rad) as accessory cells (AC). Naïve and memory CD8^+^ T cells were labeled with Carboxyfluorescein Succinimidyl Ester (CFSE, 10 μM, Thermofisher Scientific) for 15 min at 37 °C and then washed twice with RPMI-1640 plus 10% FBS. Cells were cultured in vitro in U-bottomed 96-well microtiter plates at a density of 2.5×10^5^ cells/well plus same number of AC and anti-CD28 mAb (0.5 μg/ml, BD Biosciences), in 200 μl/well of complete medium (RPMI-1640), supplemented with 10% FBS (Gibco), 2 mmol/l L-glutamine (Sigma-Aldrich), penicillin and streptomycin, nonessential amino acids, sodium pyruvate (Euroclone), 50 μmol/l β-mercaptoethanol (Sigma-Aldrich). Cells were stimulated with single or pooled AEs (10 μg/ml), OVA_275-282_ peptide (10 μg/ml), or anti-CD3 mAb (1 μg/ml, clone145-2C11, BD Biosciences). After 24 hr of incubation at 37 °C, cells were stimulated for further 72 h with IL-2 (100 IU/ml, Roche). Finally, proliferation was measured with flow cytometry as CFSE dilution in daughter cells.

### IFN-γ ELISpot

Lymphocytes obtained from spleen or CNS of mice with EAE or naïve mice were stimulated with 17 peptide pools each containing 7-9 AEs at 10 µg/ml (arranged in a matrix-based combination) and then tested by enzyme-linked immunospot (ELISpot) assay. Briefly, 96-well high-affinity plates (Millipore Corporation, Bedford, MA, USA) were coated with 10 µg/ml of capture mAb against IFN-γ (BD Biosciences) at 4 °C overnight. Plates were blocked for 2 hr with blocking solution (PBS containing 2% BSA). A total of 10^5^ cells were added to each well and stimulated for 18 hr with peptides. Biotinylated anti-IFN-γ (BD Biosciences) diluted to 5 µg/ml in blocking solution was added and incubated for 2 hr in 5% CO_2_ at 37 °C. Plates were washed and incubated with alkaline phosphate (AKP)-streptavidin (BD Biosciences) and developed with Sigmafast BCIP/NBT (Sigma). The reaction was stopped by rinsing the plate with distilled water. Each well was counted by an ELISPOT reader system (AELVIS reader system).

### IFN-γ ELISA

Splenocytes (5x10^5^cells/well) were seeded in 96-well U-bottomed microtiter plates in 200 μl of medium (RPMI-1640, Gibco) supplemented with 10% fetal bovine serum (FBS, Gibco), 2 mmol/l L-glutamine (Sigma-Aldrich), penicillin and streptomycin, nonessential amino acids, sodium pyruvate (Euroclone), 50 μmol/l β-mercaptoethanol (Sigma-Aldrich). Cells were stimulated with MYH9_761-768_ peptide (10 μg/ml) or OVA_275-282_ peptide (10 μg/ml); as positive control, cells were stimulated with anti-CD3 mAb (1 μg/ml, clone 145-2C11, BD Biosciences); as negative control, medium only was used. Plates were incubated for 48 hr at 37 °C in a 5% CO_2_ humidified incubator. Thereafter, supernatants were collected and frozen at −80 °C until ready for cytokine measurement. IFN-γ was quantified using anti-mouse OptEIA ELISA Set for interferon (BD Biosciences, San Diego, USA).

### Statistics

Statistical analyses were performed using GraphPad Prism version 9. In all figures, the results are expressed as mean ± standard error of the mean (SEM). The Mann-Whitney test was used to compare experimental groups in all experiments. Significant outliers were excluded with Rout test. Correlation between variables was calculated with Spearman correlation. In all in vivo experiments, at least 4 mice per group were used; in in vitro experiments, each condition was tested in 3-6 replicates. No statistical methods were used to for preliminary sample size estimate. Mice were randomly allocated into experimental groups and were assessed blindly. Every experiment was repeated at least twice. The magnitude of the *P* value is represented with asterisks according to the following legend: *P < 0.05, **P < 0.01, ***P < 0.005.

## Supplementary information

Supplemental figure legends

Supplemental table

Supplemental Figure 1

Supplemental Figure 2

Supplemental Figure 3

## Data Availability

All data generated or analyzed during this study are included in this published article and its supplementary information files.
