# Supplementary material for: CD8+ T cells specific for cryptic apoptosis-associated epitopes exacerbate experimental autoimmune encephalomyelitis
Source: Cell Death Dis. 2021 Oct 29;12(11):1026. doi: 10.1038/s41419-021-04310-6 (PMC8556378; doi:10.1038/s41419-021-04310-6)
Supplement: Supplementary file 1 — Supplemental figure legends [file 41419_2021_4310_MOESM1_ESM.docx]

SUPPLEMENTAL FIGURE LEGENDS

*Figure S1. IFN-γ ELISpot after challenge with a peptide matrix reveals immunodominant AEs.*

C57BL/6 mice were immunized with MOG_35-55_ to induce EAE (n=5); as controls (CTRL), not immunized mice were used (n=6). Mice were sacrificed at day 28 and splenocytes were challenged ex vivo for 18 hours with 17 peptide pools each containing 7-9 AE-peptides at 10 µg/ml (arranged in a matrix-based combination), then IFN-γ ELISpot was performed. As positive control, cells were stimulated with MOG_35-55_ peptide. **(A)** Violin plots showing the number of spots per 10^6^ splenocytes from single mice. The pools inducing higher response in MOG_35-55_-immunized compared to control mice are highlighted with red frames. **(B)** Scheme of the peptide matrix. The cell color indicates the restriction and the protein of each peptide. The five peptides that are shared among the pools giving positive results are highlighted in red. **(C)** Table showing the sequences and the percentile rank according to IEDB of the five identified peptides. The three selected are indicated in red.

*Figure S2. AE-specific CD8*^+^ *T cells display an effector/memory phenotype in the CNS of mice with EAE.*

Active EAE was induced in C57BL/6 mice and spleens (SPL) and central nervous system (CNS) were collected after 28 days. **(A)** Gating strategy that was used for the flow cytometry analysis of AE-specific CD8^+^ T cells. **(B)** Histograms showing the expression of the indicated markers in total (black) or dextramer^+^ (red) cells, extracted from the SPL (empty) or the CNS (filled) of mice with EAE. Numbers indicate the geometric mean fluorescence intensity.

*Figure S3. AE-specific CD8*^+^ *T cells can be found in the thymus of naïve mice.*

Thymocytes from C57BL/6 naïve mice (n=2) were analyzed by flow cytometry. **(A)** Representative stainings and **(B)** cumulative analysis of CD8^+^ T cells specific for the indicated single peptides, in gated CD4^+^ CD8^+^ double positive (DP) or CD4^-^ CD8^+^ single positive (SP) thymocytes. FMO, fluorescence-minus-one control.
