# Supplementary material for: CD8+ T cells specific for cryptic apoptosis-associated epitopes exacerbate experimental autoimmune encephalomyelitis
Source: Cell Death Dis. 2021 Oct 29;12(11):1026. doi: 10.1038/s41419-021-04310-6 (PMC8556378; doi:10.1038/s41419-021-04310-6)
Supplement: Supplementary file 2 — Supplemental table [file 41419_2021_4310_MOESM2_ESM.docx]

**Table S1**

List of AE-peptides.

| **Number** | **Code** | **Allele** | **Protein** | **Start** | **Length** | **Sequence** |
| --- | --- | --- | --- | --- | --- | --- |
| 1 | 1B12 | H-2-Db | MYH9 | 1308 | 9 | SQLQDTQEL |
| 2 | 1C1 | H-2-Db | MYH9 | 477 | 10 | QQLFNHTMFI |
| 3 | 1C2 | H-2-Db | MYH9 | 209 | 9 | RQLLQANPI |
| 4 | 1C3 | H-2-Db | MYH9 | 1477 | 9 | KALSLARAL |
| 5 | 1C4 | H-2-Db | MYH9 | 839 | 9 | NSIRHEDEL |
| 6 | 1C7 | H-2-Db | MYH9 | 64 | 9 | KVKVNKDDI |
| 7 | 1C8 | H-2-Db | MYH9 | 1681 | 9 | EMIQLQEEL |
| 8 | 1C9 | H-2-Db | MYH9 | 659 | 9 | ATLRNTNPN |
| 9 | 1C10 | H-2-Db | MYH9 | 862 | 10 | LAAENRLTEM |
| 10 | 1C11 | H-2-Db | MYH9 | 477 | 9 | QQLFNHTMF |
| 11 | 1C12 | H-2-Db | MYH9 | 745 | 9 | KALELDSNL |
| 12 | 1D1 | H-2-Db | MYH9 | 294 | 10 | LEPYNKYRFL |
| 13 | 1D5 | H-2-Db | MYH9 | 624 | 9 | VAGMSETAL |
| 14 | 1D7 | H-2-Db | MYH9 | 811 | 10 | VLQRNCAAYL |
| 15 | 1D8 | H-2-Db | MYH9 | 1528 | 10 | RALEQQVEEM |
| 16 | 1D9 | H-2-Db | MYH9 | 8 | 10 | KYLYVDKNFI |
| 17 | 1D10 | H-2-Db | MYH9 | 301 | 10 | RFLSNGHVTI |
| 18 | 1D11 | H-2-Db | MYH9 | 863 | 9 | AAENRLTEM |
| 19 | 1E1 | H-2-Db | MYH9 | 1019 | 9 | AKLKNKHEA |
| 20 | 1E2 | H-2-Db | MYH9 | 445 | 9 | ASFIGILDI |
| 21 | 1E3 | H-2-Db | MYH9 | 907 | 10 | TAKKQELEEI |
| 22 | 1E4 | H-2-Db | MYH9 | 1481 | 9 | LARALEEAM |
| 23 | 1E5 | H-2-Db | MYH9 | 1101 | 9 | MALKKIREL |
| 24 | 1E6 | H-2-Db | MYH9 | 89 | 9 | LTCLNEASV |
| 25 | 1E7 | H-2-Db | MYH9 | 568 | 10 | FCIIHYAGKV |
| 26 | 1E10 | H-2-Db | MYH9 | 1713 | 9 | SSGKGALAL |
| 27 | 1E11 | H-2-Db | MYH9 | 439 | 10 | KTKRQGASFI |
| 28 | 1F1 | H-2-Db | VIM | 66 | 9 | SAVRLRSSV |
| 29 | 1F2 | H-2-Db | VIM | 22 | 10 | SRPSSNRSYV |
| 30 | 1F6 | H-2-Db | VIM | 367 | 10 | DEIQNMKEEM |
| 31 | 1F8 | H-2-Db | VIM | 353 | 10 | LEAANYQDTI |
| 32 | 1H6 | H-2-Kb | MYH9 | 120 | 8 | VINPYKNL |
| 33 | 1H8 | H-2-Kb | MYH9 | 273 | 9 | TFHIFYYLL |
| 34 | 1H9 | H-2-Kb | MYH9 | 273 | 8 | TFHIFYYL |
| 35 | 1H10 | H-2-Kb | MYH9 | 761 | 8 | VFFRAGVL |
| 36 | 1H11 | H-2-Kb | MYH9 | 119 | 9 | VVINPYKNL |
| 37 | 2A1 | H-2-Kb | MYH9 | 274 | 8 | FHIFYYLL |
| 38 | 2A2 | H-2-Kb | MYH9 | 272 | 9 | RTFHIFYYL |
| 39 | 2A3 | H-2-Kb | MYH9 | 823 | 8 | RNWQWWRL |
| 40 | 2A4 | H-2-Kb | MYH9 | 14 | 8 | KNFINNPL |
| 41 | 2A5 | H-2-Kb | MYH9 | 424 | 8 | RMFRWLVL |
| 42 | 2A6 | H-2-Kb | MYH9 | 760 | 9 | KVFFRAGVL |
| 43 | 2A7 | H-2-Kb | MYH9 | 233 | 9 | SRFGKFIRI |
| 44 | 2A8 | H-2-Kb | MYH9 | 187 | 8 | VIQYLAHV |
| 45 | 2A9 | H-2-Kb | MYH9 | 1359 | 8 | IATLHAQV |
| 46 | 2A11 | H-2-Kb | MYH9 | 823 | 9 | RNWQWWRLF |
| 47 | 2A12 | H-2-Kb | MYH9 | 826 | 9 | QWWRLFTKV |
| 48 | 2B1 | H-2-Kb | MYH9 | 186 | 9 | KVIQYLAHV |
| 49 | 2B5 | H-2-Kb | MYH9 | 1498 | 9 | LNKQFRTEM |
| 50 | 2B6 | H-2-Kb | MYH9 | 817 | 9 | AAYLRLRNW |
| 51 | 2B7 | H-2-Kb | MYH9 | 420 | 9 | ATYERMFRW |
| 52 | 2B8 | H-2-Kb | MYH9 | 641 | 9 | GMFRTVGQL |
| 53 | 2B9 | H-2-Kb | MYH9 | 603 | 9 | SSDKFVSEL |
| 54 | 2B11 | H-2-Kb | MYH9 | 1169 | 8 | VSILKKTL |
| 55 | 2B12 | H-2-Kb | MYH9 | 624 | 9 | VAGMSETAL |
| 56 | 2C1 | H-2-Kb | MYH9 | 499 | 8 | WNFIDFGL |
| 57 | 2C2 | H-2-Kb | MYH9 | 374 | 9 | VSHLLGINV |
| 58 | 2C3 | H-2-Kb | MYH9 | 642 | 8 | MFRTVGQL |
| 59 | 2C4 | H-2-Kb | MYH9 | 664 | 8 | TNPNFVRC |
| 60 | 2C5 | H-2-Kb | MYH9 | 369 | 9 | TAAQKVSHL |
| 61 | 2C6 | H-2-Kb | MYH9 | 352 | 9 | IAFKKERNT |
| 62 | 2C7 | H-2-Kb | MYH9 | 1101 | 9 | MALKKIREL |
| 63 | 2C8 | H-2-Kb | VIM | 7 | 8 | SSSSYRRM |
| 64 | 2C9 | H-2-Kb | VIM | 6 | 9 | VSSSSYRRM |
| 65 | 2C10 | H-2-Kb | VIM | 9 | 9 | SSYRRMFGG |
| 66 | 2C11 | H-2-Kb | VIM | 36 | 9 | RTYSLGSAL |
| 67 | 2C12 | H-2-Kb | VIM | 115 | 9 | ANYIDKVRF |
| 68 | 2D1 | H-2-Kb | VIM | 426 | 8 | TNLESLPL |
| 69 | 2D3 | H-2-Kb | VIM | 33 | 8 | TSTRTYSL |
| 70 | 2D4 | H-2-Kb | VIM | 62 | 9 | VTRSSAVRL |
