# Supplementary figures and images for: CD8+ T cells specific for cryptic apoptosis-associated epitopes exacerbate experimental autoimmune encephalomyelitis
Source: Cell Death Dis. 2021 Oct 29;12(11):1026. doi: 10.1038/s41419-021-04310-6 (PMC8556378; doi:10.1038/s41419-021-04310-6)

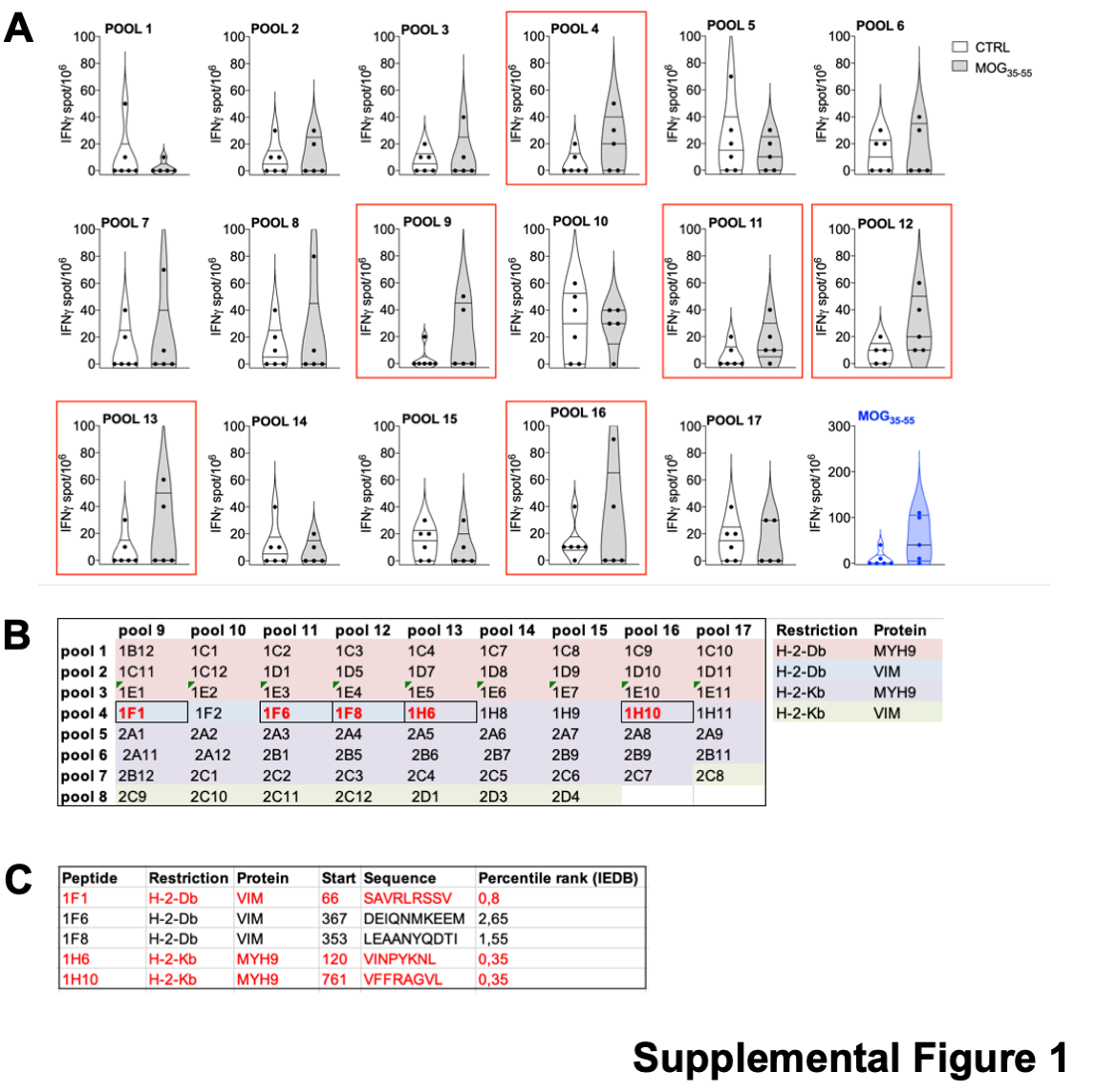

Supplement: Supplementary file 3 — Supplemental Figure 1 [file 41419_2021_4310_MOESM3_ESM.tif]

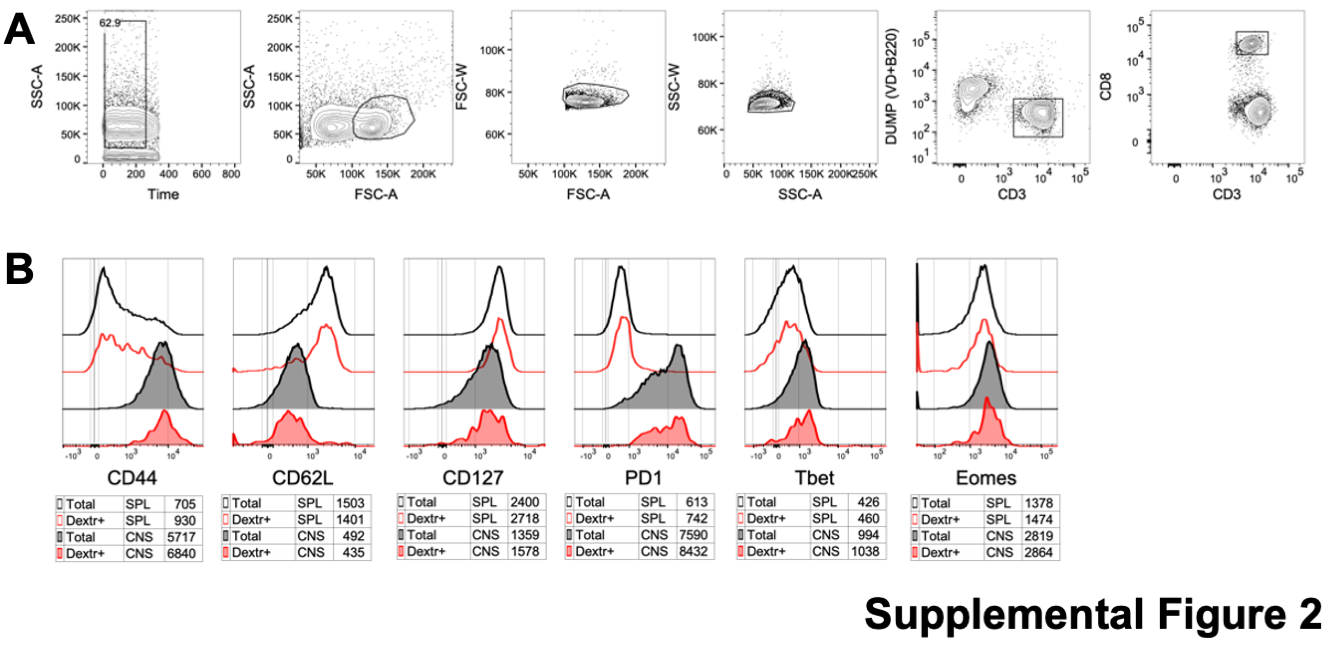

Supplement: Supplementary file 4 — Supplemental Figure 2 [file 41419_2021_4310_MOESM4_ESM.tif]

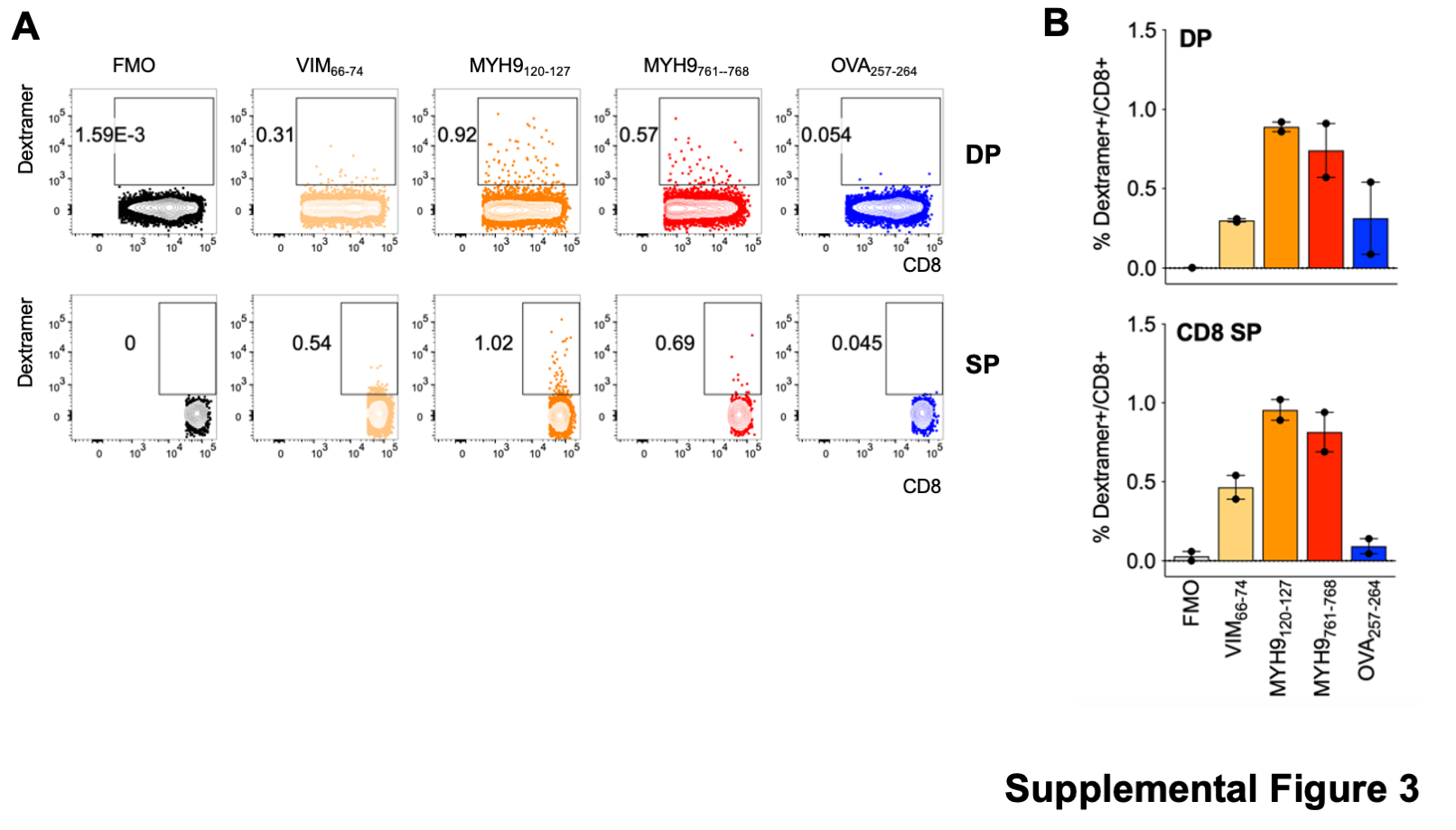

Supplement: Supplementary file 5 — Supplemental Figure 3 [file 41419_2021_4310_MOESM5_ESM.tif]
